# Abstract of a Communication from a Physician in Philadelphia, Dated December the 10th, 1801, to Dr. Patterson, in Londonderry

**Published:** 1802-04

**Authors:** 


### *Abstract of a Communication from a Physician in Philadelphia, dated December the* 10*th*, 1801, *to* Dr. PATTERSON, *in Londonderry.*

DEAR SIR,

HAVING feen two veffels advertifed for Londonderry, I eagerly embrace the opportunity to acknowledge the receipt of your letter, under date of the 25th of laft April, and to congratulate you on the return of peace to your country as well as to the reft of Europe.

I am very much obliged to you for the extracts you favoured me with from the book, entitled 'A General Hiftory of the Air,' dedicated to Dr. Mead, relative to copious venefection in peftilential fevers. Whether Mr. Webfter's Collection on the Precurfors and Caufes of Peftilential Difeafes has been anticipated by that author, as you fufpect, I cannot fay; but from the primitive abfurdity of the opinions of Mr. W. I cannot fuppofe it has been the cafe.*

The Communicator proceeds to mention Dr. Haygarth's letter, addreffed to the College of Phyficians in Philadelphia, and cites fome paffages from it, which need not be tranfcribed here, as Dr. Haygarth's tract is before the public. He particularly regrets, with Dr. Haygarth, the bad confequences which refulted from the caufe of the peftilence in 1793, being afcribed to '*Putrid coffee*' by a phyfician of eminent abilities, and he ridicules the notion of Mr. Webfter, who imputes the rife

---

* The paffage above alluded to is given on the authority of 'Clark's Examples,' where, noticing the London plague of 1665, which was imported from Holland, December, 1664, in fome bales of cotton, it is remarked, 'Of this plague died within the Bills of Mortality near 100,000. O fatal bales of cotton! Yet the true remedy was never difcovered, viz. Bleeding *ad deliquium animi*, at the firft inftant of feizure. But the reafon of this is yet a fecret.' This expedient of profufe blood-letting I have fhewn in my '*Remarks on Dr. Rush*,' publifhed in 1795, was an ancient practice in fevers, under certain provifional rules. In Britain and France, anno 1545, raged a peftilential epidemic, called *troup galant*, becaufe it chiefly feized and carried off young, ftrong, brifk people, in which *large* and *repeated* blood-letting was faid to be the only help; and I find, that, in the peftilence which prevailed in Milan, Padua, &c. in 1575, a fimilar evacuation was practifed by Palmarius. At the fame time, I obferve, that, in the *generality* of inirritative epidemic fevers, defcribed in ancient records, venefection, in every ftage and degree, was found to be pernicious. W. P.

rise of the pestilential epidemics to volcanos, earthquakes, halos, high tides, hail-stones, dead haddocks, &c. in times and countries distant from the juncture and place in which those diseases made their appearance. †

You mention, continues he, having sent a Reply to the Animadversions of the Editors of the New York Medical Repository, in expectation that you should have an opportunity of defending yourself. No such reply has yet appeared in the Repository that I have seen; and if it in any degree controverts the hypothetical doctrine of one of the Editors, I question much whether it ever will appear: For it is their practice to reject every thing unfavourable to a cause, which receives its chief support from the art of rendering all the avenues to truth impervious and remote, and the plainest facts obscure and intricate. This has been strongly exemplified with regard to the account, which they lately published, of the origin of the pestilential fever at Cadiz, in the hot season of 1800. In this account, they affirm that the disease originated in Cadiz from local causes, and had no connexion with imported contagion.

In consequence of this glaring attempt to mislead, the following paragraph appeared in the New York Gazette of the 28th of October last. 'The Conductors of the Medical Repository have given another specimen of their great partiality for such opinions and supposed facts, as in the remotest degree countenance their doctrine of the domestic origin of the pestilential fever, which of late years has occasioned such dreadful mortality in this country; while with studied caution they reject every thing that militates against it, although derived from the most authentic sources.

'In the last Number of the Repository for the present year, they have published a mutilated abstract of a Supplement to the Madrid Gazette, dated the 28th of October, 1800, relative to the

† In the 'Remarks' referred to in the preceding note, I have given reasons and instances to prove, that neither the effluvia from vegetable nor animal putrefaction can generate a specific pestilential disease; and from subsequent inquiries, I am furnished with additional arguments to confirm me in this opinion. The gentlemen, who resolutely made experiments on the matter of *black vomit*, in the yellow fever of America, received no injury; and, in the general plague of 1347-8, 'Tanners, curriers, such as cleansed bog-houses, servants in hospitals, and those employed in other nasty stinking businesses, all escaped infection; wherefore I am led to believe, that the notion of '*Putrid coffee*,' &c. wants, and ever will want, the *consent of time*. As to the fanciful causes alledged by Webster, we shall only condescend to ask, Do volcanos, showers of blood, swarms of mice or of grasshoppers, enchanted coffins, red or green crosses, handling a dead priest's bones roughly, all produce similar effects on the atmosphere, and beget the same pestilential distempers? W. P.

the origin and treatment of the fever at Cadiz; which, as it contains nothing decifive on the fubject of its introduction, but fpecifies merely the ftate of the weather previous to its occurrence, and during its prevalence, thefe gentlemen have *modeftly* conftrued into a proof of its having originated there from local caufes. But they have taken fpecial care to pafs by the facts of a later date and different complexion, publifhed in the fame paper, of the 4th of November, 1800, entitled, "Some particulars relative to the ficknefs which has depopulated Andelufia;" the contents of which follow.

'At Cadiz it blew a ftrong eafterly wind, which, paffing over a burning part of the country, augmented the exceffive heat of the fummer, whereby the atmofphere was well difpofed to receive the ficknefs, but by no means was the *caufe* of it. The greateft part of the phyficians of Cadiz were wrongfully impreffed with this opinion, and therefore applied wrong remedies, which increafed the mortality. On the 8th of Auguft, an American veffel entered the Harbour of Cadiz; the log-book of the captain mentions, that during the paffage three men died on board of the *yellow fever*. The crew, coming on fhore, went into the neighbouring ftreets and taverns, and all died of the ficknefs excepting the mate. The fever foon after fhewed itfelf among the inhabitants of the city, and there was not a houfe into which the infection did not penetrate. Terror fpread on all fides. Many of the inhabitants, not knowing that they already had the ficknefs in their bodies, fled to the Ifle De Leon, and feven other places, to the inhabitants of which thefe, perhaps, fugitive emigrants foon communicated the diforder, which was confined to the nine cities, and did not extend to Eftramadura. From the 12th of Auguft until the 1ft of November, died of this ficknefs feventy-nine thoufand five hundred perfons.'*

It did not appear in any of the fea ports of the neighbouring nations in the fame climate; of courfe, the condition of the atmofphere could have had no fhare in its generation. I have alfo been affured by Mr. Patrick Yznardi, fon to the American Conful at Cadiz, who loft feveral of his connexions by the difeafe, that it was introduced into Cadiz by a veffel from Havanna, and that the Intendant of Havanna, who was paffenger on board that veffel, had been imprifoned by order of the ma-

* In the places taken generally, the proportion of deaths was from a fourth to a fifth part of the number of inhabitants; and in Seville the mortality was fo great, that three-eighths of the people fell victims to the peftilence. W. P.

 giftrates giftrates for neglecting to inform the Commiffioners of Health of the exiftence of the diforder in the fhip; likewife, that fome of the phyficians were feverely cenfured and reprimanded for mifleading the magiftrates with refpect to the contagious nature of the diforder. This has alfo been confirmed by the late Spanifh Conful, Don Jofeph De Viar.

All the commercial towns in this country (America) efcaped the peftilential fever laft year, (1800) except Norfolk, Baltimore, and Providence; the former about 400 miles fouth, and the latter 291 miles north eaft of Philadelphia. In the prefent year they all have efcaped, except Norfolk and New York; and the latter was affected only partially, owing to the advanced period of the warm feafon when it occurred. Thefe circumftances, with the circumftances attending its prefence in former years, and the fact admitted by *all parties*, that it never has been obferved to make its firft appearance in the moft marfhy and unwholefome fituations, remote from the fea port towns, even in the fouthern parts of this country, where, it is acknowledged by every one, that the remote or efficient caufe of bilious or remitting fevers is moft abundant, are fatisfactory proofs that it is not derived from the fame caufe, and baffle all pretenfions to explanation on the principle of an epidemic or peftilential conftitution of the atmofphere.

It muft appear furprifing to you, that difputes, with regard to the moft efficacious methods of treating the peftilential fever, fhould ftill exift among the phyficians of this country, as well as among the Medical Gentlemen in the Weft Indies. This, I believe, can only be accounted for, from the unfortunate propenfity in phyficians to theorife, rather than to obferve. The fondnefs for a favourite difcovery, I fufpect, has mis-led the judgment of Dr. Chisholm, with refpect to the power of Mercury; and the diffatisfied mind of Dr. Jackfon has led him to change his opinion relative to the nature and caufe of this deftructive difeafe. His pages teem with the fpirit of difcontent and oppofition. Dr. Chisholm has detailed many of his facts in fuch a loofe, incoherent, and flovenly manner, and has viewed objects fo much through the vague and unfettled medium of *theory*, in his new edition, that he has greatly diminifhed the weight of his authority.

The employment of mercury in fmall and repeated dofes, for the purpofe of fpeedily exciting falivation, is the fafhion of the day in this country, as well as in the Weft India Iflands, not only in the peftilential yellow fever, but in almoft all other difeafes, even in thofe of an oppofite nature. This new and incongruous practice is defended on the principle taught by the late John Hunter, that two actions of different force cannot exift in in the human fyftem at the fame time. By introducing a ftronger power, therefore, into the fyftem, they expect to difarm the weaker, and thereby to fubdue the difeafe, which they confider to be only an affemblage of difordered action; confequently, they direct all their views to create an artificial difeafe in the mouth and fauces as fpeedily as poffible, that the ftronger action thus produced may overcome the weaker. But if any dependance could be placed on this principle, pain and inflammation excited by other means would have the fame effect, and every variety of fever might be cured by covering the whole body with bliftering plafters, or wetting it with cauftic fluids, and giving ardent fpirits and fpices internally; which, according to the principles taught by Brown, would wear down the excitability to the healthy point.

Relying on the validity of this principle, fome have even employed mercury in the cure of phthifis pulmonalis, in oppofition to the dictates of common fenfe, and the experience of ages.

In the few cafes of acute rheumatifm, in which I have feen a falivation induced, it has invariably aggravated the fever, and rendered the pain intolerable; and there is reafon to believe, that mercury has a fimilar effect in all other inflammatory affections in their acute ftate, excepting when it operates freely as a purgative.*

I have feen numerous inftances of patients with intermittent fever who have been falivated, without any fenfible effect being produced on the fever. During the exacerbation of the difeafe, the flow of faliva was generally fufpended, but it flowed again during the intermiffion. A fimilar fact was obferved by Granger during the campaign in Flanders, in 1746 or 7. This affords a ftrong argument to prove, that falivation does not occafion a ceffation of the febrile fymptoms of peftilential fevers; but, on the contrary, that the flow of faliva is owing to the ceffation, or folution of the fever.

---

* Thefe ftrictures of my worthy Correfpondent, whofe practical authority I hold in high eftimation, muft be received with confiderable limitation in *this country*, as, if fully admitted, they would tend to deter phyficians from the ufe of mercury, in any form or degree, in cafes of acute rheumatifm; whereas, my experience leads me to approve of its exhibition, not only in the inflammatory ftate of this difeafe, but in pleuritic cafes even whilft the ftitch is fharp; and at the moment of writing this note, I have under treatment a cafe of the former malady, wherein fmall and repeated dofes of mercury, combined with opium and antimonial tartar, have produced a manifeft good effect. For fome time paft, indeed, practitioners in thefe iflands have found mercury, thus affociated, to be fignally curative in inflammatory affections. W. P.

The action of mercury on the salivary glands never removes the eruptive fever of Small-pox, nor diminishes the number of pustules. In a recent case of Measles, salivation had no effect in preventing the eruption; but it certainly augmented the fever, and added to the distress of the cough as well as of other inflammatory symptoms, by occasioning a painful inflammation of the gums and tonsils.

The Measles, which were imported to New-Castle and Wilmington on the Delaware, in the summer of the present year, by some vessels from Ireland, on board of which a great many children had died during the voyage, soon after their appearance in those places, broke out in this city, and were first observed in a school at the southern extremity of the town, kept by Mrs. Watson, where upwards of twenty of the scholars were attacked within two or three days of each other, though it was not known that any person had come into the school with the disease, or who had lately recovered from it. These scholars, residing in different parts of the town, communicated the disease to others, and so on in succession, till it has now become epidemic or general throughout the city. This is contrary to the observations of Sydenham, who confines its rise and progress to the vernal season; and says, it declines after the summer solstice as rapidly as it increases previous to that period.

If the masters of the vessels from Ireland had followed the example of many of those from the West Indies, and had denied the existence of the Measles on board their ships, physicians, who are more accustomed to speculate than to observe, would doubtless have ascribed the appearance of the disease, in this city this year, to an epidemic morbillous constitution of the atmosphere, and probably would have imputed the absence of the pestilential Yellow Fever to the superior force of the Measles in occupying the air, and banishing all other distempers from the sphere of its dominion, or compelling them to do homage to it, by wearing its livery. But the facts on this occasion are too clear to give any countenance to such a subterfuge. If the pestilential constitution prevailed, the Measles, in the majority of cases, should have been accompanied with pestilential symptoms, but it has not been so; on the contrary, in the majority of cases, the symptoms have been very mild.

In consequence of the respectable testimony in favour of the superior advantages to be derived from substituting the Kine-pox, for the purpose of banishing the Small-pox from the catalogue of diseases, Dr. Waterhouse introduced it last year into Cambridge, near Boston; and, in a considerable number of persons whom he inoculated with the matter of Kine-pox, and afterward afterward with the virus of Small-pox, it has fupported the character which it has acquired in the united kingdom of Great Britain and Ireland.

The generality of the phyficians in this city alfo propofe to fubftitute it in the enfuing fpring. It has already been introduced here, and is making progrefs; fo that we fhall be able to procure a fufficiency of matter for the purpofe. I fhould have inoculated with the matter of Kine-pox two years ago, having received an infected thread from Dr. Pearfon; but I was deterred at that time, from the fact mentioned by Dr. Jenner, of a perfon being liable to be infected with the Kine-pox more than once, though it rendered him for ever fecure againft taking the Small-pox. To fubftitute the Kine-pox, therefore, for the Small-pox on thefe conditions, appeared to me too much like exchanging a temporary evil limited in extent, for one of frequent occurrence, and of which we fhould be in perpetual danger.* But the proofs of the fuperior mildnefs of the Kine-pox, and the great difficulty and extreme little chance of ever taking it in the natural way, lately collected and publifhed by Jenner, Ring, Lettfom and Aikin, together with the candid and fummary ftatement of Dr. Pearfon in Duncan's Annals of Medicine, for the year 1800, have removed all my apprehenfions, and of courfe all my objections to it.

Mr. Lee, a medical ftudent in the Pennfylvania Hofpital, has attempted (and in my opinion with fuccefs) to refute the hypothefis of Dr. Mitchill refpecting the gafeous oxyd of fepton, or in plain Englifh, the nitric acid, in a volatile or gafeous ftate, by the following facts and arguments.

'Nitrous air never exifts in the atmofphere, but is inftantly converted into nitrous acid. The nitrous acid moreover has never been found in the atmofphere in a free ftate, but is always the product of putrefaction; and as it is generated, it unites with other bodies, particularly with ammonia. This truth Dr. Mitchill himfelf acknowledges, although he ftill believes that nitrous acid is the caufe of peftilential difeafes. But if it fhould be conceded that the nitrous acid is fometimes found perfectly free in the atmofphere, it cannot retain its freedom long enough, efpecially in cities, to act as a caufe of general complaint, becaufe it would very foon combine with other bodies. The fubftance for which it has the greateft affinity is the vegetable alkali, next to this the foffil alkali, then barytes, and afterward

* All grounds for fuch apprehenfion are removed by knowing, that the Vaccine difeafe is *not infectious* or communicable through the medium of the air and refpiration, but that its virus muft be *inoculated*, in order to impart it to the human fubject. W. P.

afterward lime or calcareous earth. As the firſt three are not very abundant in cities, lime would be the ſubſtance with which it would form the chief combination.

'Calcareous earth is very plentiful in the cities of the United States, as it forms a very conſiderable part of the buildings. If therefore the gaſeous oxyd of ſepton* exiſted in the atmoſphere of cities, a union would neceſſarily take place between it and the lime; but that no ſuch union takes place is evident, becauſe every chemiſt knows that the ſalt formed by theſe two ſubſtances (nitrate of lime or nitrous ſelinite) is readily ſoluble in water, and very deliqueſcent. If the fact was otherwiſe, the houſes would ſoon fall to pieces. It is clear, therefore, from theſe conſiderations, that the nitrous acid does not exiſt in the atmoſphere, and that it cannot be the cauſe of peſtilential or other epidemic fevers.

'If the acid of putrefaction, or nitrous acid gas, was the cauſe of peſtilence, how comes it that chemiſts breathe nitrous vapours in their laboratories for hours together with impunity? And that phyſicians adminiſter to their patients large quantities of nitrous acid without the leaſt injury?

'The nitrous acid is never converted into a permanent gaſeous ſtate by the heat of the ſun in the hotteſt climate on earth; neither is the muriatic acid. To give theſe two acids aeriform elaſticity, a conſiderable degree of heat, together with the agency of a third body, is neceſſary.'†

That no ſuch gas as that called by Dr. Mitchill, Gaſeous Oxyd of Septon, exiſts in, or can be derived from, the perſpirable matter, is fully proved by the judicious experiments and obſervations of Mr. Abernethy. For theſe experiments demonſtrate, that the perſpirable matter conſiſts entirely of Carbonic gas and Azotic gas, in the proportion of a little more than

---

* "This is preciſely the ſame ſubſtance as Carm. Smyth's nitrous vapour, employed in the naval and military hoſpitals with ſuch ſucceſs in deſtroying the cauſe of fever." American note.

† They who are in danger of being entangled in the perplexities which involve the hypotheſis of *Septon*, (azote or nitrogene,) or ſimilar ſpeculations, will find an excellent admonition contained in the following plain and elegant language of the preſent Prof. Gregory: "Antiquum enim et uſitatiſſimum apud medicos eſt, ſibi aliiſque perſuadere ſe rem intelligere, cum nomen tantum didicerint, novamque rem ſe credere inveniſſe, cum novum tantum rei antea bene notæ nomen impoſuerint. Caveant autem medici, caveant imprimis medicinæ ſtudio incumbentes, ne hoc modo ſibimet ipſis dent verba, umbram que pro ſubſtantia captent.") (*Conſpectus, &c. Auditoribus ſuis, p. 7.*) However, Mr. Davy having found, that gaſeous oxyd of azote is reſpirable when perfectly free from nitrous gas, and appears to ſupport life longer than common air, the ſeptic theory of contagion, as this ingenious chemiſt obſerves, is of courſe completely overturned. W. P.

than two-thirds of the former to fomething lefs than one-third of the latter.†

Dr. Chifholm has alfo combated the doctrine, from the circumftance that putrefiable materials of marfhes do not afford a fufficient quantity of azote for the formation of nitrous acid, either in a fixed or volatile ftate.

Dr. Ramfay, of Charlestown, has publifhed an Oration, which he delivered before the Medical Society of that city, the 1ft of January laft, "On the Improvements in Medicine in the Courfe of the Eighteenth Century," wherein he has acquitted himfelf very handfomely, though the limits of the Oration have compelled him to treat the fubject too fuperficially to make it very interefting, except as a memorandum of names and dates.

Dr. Barnwell, a native of Ireland fettled in this city, whofe opportunities of making obfervations in different climates have been confiderable, and who appears to be a man of erudition, has now in the prefs a volume, entitled "Sketches of Medical Geography, &c."

A Compendium of Chemiftry, by J. Parkinfon, of London, with an Appendix, containing an Account of the principal Objections to the Antiphlogiftic Syftem of Chemiftry, by Dr. Woodhoufe, Profeffor of Chemiftry in the Univerfity of Pennfylvania, is alfo printing in this city.

The fecond Number of the fifth Volume of the New York Repofitory, due laft month, has not yet made its appearance; owing, I believe, to Dr. Mitchill, one of the Editors, being elected a Member of Congrefs, and being under the neceffity of attending the feffion (which takes place this month) at the Federal City.

Thofe fingular infects, called *Cicadæ Americanæ*, or locufts, which make their appearance in this country regularly every feventeen years, and come from a great depth beneath the furface of the ground, paid us their periodical vifit laft year in numerous fwarms; but, from the luxuriance of vegetation, they did no material damage, except to the tender foliage of the foreft and a few fruit trees.

Notwithftanding

† The conftituent principles of the cutaneous perfpirable matter, according to Plenck, are water, animal gas or carbonated hydrogene, and azotic gas. Sweat, according to the fame author, confifts of cutaneous perfpirable matter, glandular fmegma, and the ferum of the blood. The fmegma is an oily, febaceous fubftance; and the ferum of the blood contains water, albuminous gluten, jelly, aerated foda, and culinary falt. The property of changing blue vegetable juices red, which is afcribed chiefly to the fweat of gouty people, is faid to proceed from phofphoric acid. W. P.

Notwithftanding the great mortality which Philadelphia and New York have experienced of late years, in confequence of the peftilential yellow fever, the population has increafed amazingly in both. In the year 1791, the number of inhabitants in Philadelphia, including the northern and fouthern liberties, did not exceed 52,000; this year, according to the late cenfus, they exceed 72,000. New York has increafed ftill more rapidly; the number of its inhabitants, at prefent, exceeding 50,000, which is almoft double the number they were ten years ago.

The many interruptions to which a practical phyfician is conftantly fubject, muft plead an apology for the inaccuracies, &c. committed by, Your's, &c.

M. D.

---

*** As it is probable, from what is obferved in the preceding pages, that the Bar of the New York Repofitory will be fhut againft Dr. Patterfon's defence, and that he will be obliged to apply for a hearing to the judicatory of the London Medical Journal; he relies on the candour of the Editors of this impartial print, that they will not follow the example of the American Editors, who feem to have ufurped the privilege of the Hibernian tribunal, with whom it is faid to be the fafhion, "to *condemn* a man firft and to put him on *trial* afterwards."

